# Secretory Activity Is Rapidly Induced in Stigmatic Papillae by Compatible Pollen, but Inhibited for Self-Incompatible Pollen in the Brassicaceae

**DOI:** 10.1371/journal.pone.0084286

**Published:** 2013-12-26

**Authors:** Darya Safavian, Daphne R. Goring

**Affiliations:** 1 Department of Cell and Systems Biology, University of Toronto, Toronto, Ontario, Canada; 2 Centre for the Analysis of Genome Evolution and Function, University of Toronto, Toronto, Ontario, Canada; Iowa State University, United States of America

## Abstract

[In the Brassicaceae, targeted exocytosis to the stigmatic papillar plasma membrane under the compatible pollen grain is hypothesized to be essential for pollen hydration and pollen tube penetration. In contrast, polarized secretion is proposed to be inhibited in the stigmatic papillae during the rejection of self-incompatible pollen. Using transmission electron microscopy (TEM), we performed a detailed time-course of post-pollination events to view the cytological responses of the stigmatic papillae to compatible and self-incompatible pollinations. For compatible pollinations in *Arabidopsis thaliana* and *Arabidopsis lyrata*, vesicle secretion was observed at the stigmatic papillar plasma membrane under the pollen grain while *Brassica napus* stigmatic papillae appeared to use multivesicular bodies (MVBs) for secretion. Exo70A1, a component of the exocyst complex, has been previously implicated in the compatible pollen responses, and disruption of Exo70A1 in both *A. thaliana* and *B. napus* resulted in a loss of secretory vesicles/MVBs at the stigmatic papillar plasma membrane. Similarly, for self-incompatible pollinations, secretory vesicles/MVBs were absent from the stigmatic papillar plasma membrane in *A. lyrata* and *B. napus*; and furthermore, autophagy appeared to be induced to direct vesicles/MVBs to the vacuole for degradation. Thus, these findings support a model where the basal pollen recognition pathway in the stigmatic papilla promotes exocytosis to accept compatible pollen, and the basal pollen recognition pathway is overridden by the self-incompatibility pathway to prevent exocytosis and reject self-pollen.

## Introduction

During the initial stages of pollen-pistil interactions in the Brassicaceae, a basal pollen recognition pathway is activated to allow compatible pollen grains to adhere and germinate on the receptive stigmatic surface of the pistil. The stigma can also function as a barrier to prevent inappropriate pollen grains, such as foreign pollen or self-incompatible pollen, from germinating on the stigmatic surface (reviewed in [1], [2], [3]). The surface of the stigma is covered with stigmatic papillae that are coated with a continuous waxy cuticle layer overlaid by proteinaceous pellicle, and these layers are important for the initial pollen contact [4], [5], [6]. Following pollen capture by the stigmatic papilla, the exterior pollen coat is mobilized towards the contact site where a ‘foot’ is formed, by the mixing of lipids and proteins from the pollen coat with the stigmatic papillar surface [7], [8]. For a compatible pollen grain, hydration follows, and the stigmatic papilla plays a fundamental role in this step by providing the pollen grain with water for hydration [9], [10], [11]. Following this, the pollen tube emerges and enters the stigma by growing through the expanded cell wall of the stigmatic papilla [2], [5], [6]. The pollen tube traverses down to the base of the stigma, enters the transmitting tract, and then continues to an ovule for fertilization. For self-incompatible pollen, rejection occurs swiftly and the initial stages of pollen hydration and pollen tube penetration into the stigma do not take place [12]. Thus, the pollen discrimination system found in the Brassicaceae rapidly takes place as a result of the reliance of pollen grains on the stigmatic papillae for these early events (reviewed in [1], [2]).

With compatible pollinations, very little is known about the factors mediating the basal pollen recognition pathway in the stigmatic papilla. Two stigma-specific secreted glycoproteins in *Brassica*, the S-locus glycoprotein (SLG) and the S-locus Related-1 protein (SLR1) are proposed to mediate the process of pollen adhesion [13], [14], potentially through interaction with the small pollen coat proteins, PCP-A1 and SLR1-BP, respectively [15], [16]. Exo70A1, a subunit of the exocyst, is also required as part of the basal pollen recognition pathway in *Arabidopsis* and *Brassica* stigmas to promote pollen hydration and pollen tube penetration of the stigmatic surface [17]. The factors that initiate the rejection of self-incompatible pollen are well-defined and are encoded by two polymorphic genes: the *S-locus Cysteine-Rich/S-locus Protein 11* (*SCR/SP11*) gene and the *S Receptor Kinase* (*SRK*) gene. Self-incompatible pollen is rejected when *S* haplotypes match, and the allele-specific pollen SCR/SP11 ligand binds and activates the corresponding stigma SRK (reviewed in [18], [19], [20]). Two proteins have been identified as functioning downstream of SRK in the stigma: the *M* locus Protein Kinase (MLPK) in *B. rapa* [21], [22] and the ARM-Repeat Containing 1 (ARC1) E3 ubiquitin ligase in *B. napus* and *A. lyrata* [23], [24], [25], [26].

We have previously proposed that Exo70A1 functions in the stigma at the junction of the compatible and self-incompatible pollen responses where it is required for the basal pollen recognition pathway and inhibited by the self-incompatibility pathway [17], [27]. Exo70A1 is essential for growth and development as A. *thaliana exo70A1* mutants are stunted in growth with a number of defects in processes such as polar cell elongation [28], pectin deposition in the seed coat [29], cytokinesis and cell plate maturation [30], recycling of auxin efflux carriers for polar auxin transport [31], and the development of tracheary elements [32]. Exo70A1 has also been shown to be localized to the tip of growing pollen tubes [33]. In our previous study [17], a specific role for Exo70A1 in the basal compatible response was established in *B. napus* when transgenic *B. napus* plants were generated expressing an *Exo70A1* RNAi construct controlled by the SLR1 promoter (which drive strong stigma-specific expression in the late stigma stages approaching flowering [34], [35], [36]). The *B. napus* SLR1 promoter:*Exo70A1* RNAi transgenic plants were completely wild-type plants in appearance, including the stigmatic papillae, but the stigma-specific suppression of Exo70A1 expression caused compatible pollinations to be unsuccessful [17]. The basal compatible response was absent in *A. thaliana exo70A1* elongated stigmatic papillae as wild-type Col-0 pollen failed to hydrate on these mutant papillae, and this specific defect was rescued by the expression of an SLR1 promoter driven *RFP-Exo70A1* construct [17].

In response to compatible pollen, the basal pollen recognition pathway is proposed to assemble the exocyst complex with Exo70A1 to tether post-Golgi secretory vesicles to the stigmatic papillar plasma membrane underneath the pollen contact site. The secretory vesicles are thought to discharge cargo to the apoplastic space to promote pollen hydration and pollen tube penetration of the stigma surface. Consistent with this model, vesicle-like structures have been observed in the *B. oleracea* stigmatic papillar cell wall following pollination or treatment with compatible pollen coating [2], [37], [38]. In addition, calcium spikes, actin polymerization, and microtubule depolymerization have been detected in stigmatic papillae as part of the basal pollen recognition pathway for compatible pollen [39], [40], [41]. Self-incompatible pollen has been proposed to be rejected by inhibiting the basal compatible pollen response [2], [17], [27], [42]. This is thought to occur by the activation of the SRK signaling pathway in response to self-incompatible pollen, followed by the inhibition of Exo70A1 by ARC1, leading to a dysfunctional exocyst complex and disruption of vesicle secretion [17], [27]. We have previously shown that Exo70A1 binds to and is ubiquitinated by ARC1 using *in vitro* assays, and the expression of the SLR1 promoter:*RFP-Exo70A1* construct in transgenic *B. napus* plants caused a partial breakdown of self-incompatibility. Significantly, the rejection of compatible pollen by the *B. napus* SLR1:*Exo70A1* RNAi transgenic stigmas mirrored the self-incompatible pollen rejection response [17]. In the present study, we have set out to further test these models by investigating the presence or absence of secretory vesicles in the stigmatic papilla, following compatible and self-incompatible pollinations in *Arabidopsis thaliana*, *Arabidopsis lyrata* and *Brassica napus*. Altogether, our results support our proposed model for the regulation of secretion leading to compatible pollen acceptance or self-incompatible pollen rejection in the Brassicaceae.

## Materials and Methods

### Plant material and growth conditions

The *Arabidopsis* species used in this study were wild-type self-compatible *A. thaliana* Col-0, and wild-type self-incompatible *A. lyrata* ssp. *petraea* from Northern Sweden [43]. The *Brassica napus* cultivars used in this study were Westar which is self-compatible, and W1 which is self-incompatible (W1 carries the S_910_ haplotype introgressed into the Westar background; [44]). The *A. thaliana* Col-0 *exo70A1-1* mutant, transgenic *B. napus* Westar *Exo70A1* RNAi R2 plants, and transgenic *B. napus* W1 *RFP:Exo70A1* S1 and S2 plants were also examined [17]. All plants were grown in growth chambers under long-day conditions consisting of a 16 hour light/8 hour dark photoperiod at 22°C. The *A. lyrata* plants required vernalization to induce flowering, and cross-compatible pollinations were carried out by crossing individuals from the P6 and P7 populations [43]. *A. thaliana* Col-0 seedlings were grown on plates to examine clathrin-coated endocytic vesicles in root tips, and roots were harvested at day 6 as described by Dhonukshe *et al* [45].

### *A. thaliana*, *A. lyrata* and *B. napus* crosses for TEM analysis

For all pollinations, humidity levels were carefully monitored and were in the 20–40% relative humidity range as high humidity can cause pollen hydration, bypassing the basal compatible response or self-incompatibility [11], [42], [46], [47], [48], [49], [50], [51]. When grown under increased humidity conditions, the *exo70A1* mutant was recently found to have increased pollen tube growth and seed production following hand pollinations (CH Liu, personal communication [32]). For both *A. thaliana* Col-0, and the *A. thaliana* Col-0 *exo70A1-1* mutant, pistils were emasculated at the final bud stage of flower development (stage 12 [52]), and covered in plastic wrap for 24 hours to allow the stigmatic papillae to mature and prevent dehydration prior to sampling. Subsequently, the plastic wrap was removed, and the pistils were hand-pollinated by removing anthers from freshly opened *A. thaliana* Col-0 flowers with a pair of fine forceps and gently brushing 1–4 anthers across the stigmatic surface. For the *A. lyrata* crosses, the optimal timing for pistil receptivity and pollen viability were different, and pistils from 1–2 day old open flowers were hand-pollinated with pollen from 2–3 day old open flowers [24]. For the E-64 inhibitor treatment, *A. lyrata* inflorescences were first incubated for one day in 2 ml of ½ Murashige and Skoog liquid culture media with 3% sucrose and 100 µM E-64 [53] to take up the inhibitor, and then open flowers were self-pollinated. For the *B. napus* Westar, W1, Westar *Exo70A1* RNAi R2, and W1 *RFP:Exo70A1* plants, flower buds that were just opening up were emasculated, covered in plastic wrap, and then pollinated 24 hours later using anthers from fully opened flowers.

For all crosses, pollinated pistils were harvested at the specific time-points between 5 and 20 min after pollination. For the compatible pollinations, each experiment involved five different stigmas, and five stigmatic papillae/stigma were examined (n = 25). For unpollinated stigmatic papillae, self-incompatible *A. lyrata* and *B. napus* pollinations, the E-64 treated *A. lyrata* pistils, *A. thaliana* Col-0 *exo70A1-1* mutant, transgenic *B. napus* Westar *Exo70A1* RNAi R2 line, and the transgenic *B. napus* W1 *RFP:Exo70A1* S1 and S2 plants, five different stigmas and two stigmatic papillae/stigma were examined (n = 10).

### TEM Analysis

All harvested pistils were immediately fixed upon removal in 25% glutaraldehyde and 2% paraformaldehyde in 0.2 M phosphate buffer (pH 7.2) for 3 hours at the room temperature under vacuum to allow thorough penetration of fixative, a method demonstrated to preserve pollen-papilla interface and avoid artifacts, equivalent to osmic vapour technique described by Elleman and Dickinson [54]. The pistils were then rinsed in three changes of phosphate buffer and post-fixed in buffered 1% OsO4 for one hour. Following fixation, dehydration was carried out through an ethanol series (30%, 50%, 70%, 80%, 90% and 100% EtOH), and then pistils were embedded in Spurr's resin (modified from [8]). To find the pollen-stigma interface, serial sections were taken from specific areas of the embedded pistil and examined by light microscopy observations of the thick sections. From the area of interest, identified by thick sectioning, ultrathin sections of 80–90 nm thickness were cut with a Reichert Ultracut E ultra-microtome using a diamond knife. The serial sections were gathered on copper grids, and post-stained with uranyl acetate and lead citrate for 40 and 3 minutes, respectively. Longitudinal sections were examined and photographed with a Hitachi H-7000 TEM at 75 Kv. The same procedures were followed to fix and examine the *A. thaliana* seedling root tips.

### Labeling of autophagosomes with monodansylcadaverine (MDC)

*A. lyrata* pistils were pollinated with either self-incompatible pollen or cross-compatible pollen, removed at 10 min post-pollination, and fixed in 3∶1 Ethanol: Glacial acetic acid for 15 min to try to retain pollen attached to the stigmatic papillae. The pistils were then washed twice with 90% ethanol for 15 min each time and incubated in chloral hydrate in 30% glycerol for several hours to clear the tissue. Finally, the fixed and cleared stigmas were incubated with 0.05 mM MDC in phosphate buffered saline (PBS) at room temperature for 15 min [55]. After incubation, stigmas were washed four times with PBS, mounted in Vectashield (Vector Laboratories, H-1000) to prevent photobleaching and viewed using a Zeiss Axioskop2 plus fluorescence microscope. A minimum of five pistils were stained, mounted and examined for the self-incompatible pollinations or the control cross-compatible pollinations.

### Using GFP:ATG8a to detect autophagosomes in transgenic *A. lyrata*

The GFP:ATG8a construct [56] was used to transform *A. lyrata* plants through *Agrobacterium*-mediated floral dip transformation [57], and followed by hand pollinations with cross-compatible pollen [24]. The transformed T_0_ seeds were selected for by spraying with 0.1% Basta, following by genotyping with Basta primers (5′AGGCCAATAACAGCAACCAC3′ and 5′CGGAGAGGAGACCAGTTGAG3′). For visualizing GFP fluorescence, freshly opened flowers from wild-type and transgenic GFP:ATG8a plants were emasculated and pollinated with wild-type *A. lyrata* pollen as described. At 10 min self-incompatible and cross-compatible pollinations, stigmas were removed, mounted in Vectashield (Vector Laboratories, H-1000), and observed using LSM510 confocal microscope.

## Results

### Basal pollen recognition responses in stigmatic papillae from *A. thaliana*, *A. lyrata* and *B. napus*

The surface of the stigma is covered with stigmatic papillae that come in contact with pollen grains, and pollen hydration is one of the earliest visible signs that a compatible pollen grain has been accepted on *Brassicaceae* dry stigmas. In our previous work, both *A. thaliana* and *B. napus* pollen grains were found to have hydrated in the first 10 minutes following pollen application [17]. Exo70A1 was also required in the stigmatic papillae for the basal pollen recognition pathway to promote compatible pollen hydration, and so the proposed vesicle secretion would be predicted to be visible during this time [17]. Thus, time points of 0, 5, 10, and 20 minutes were selected for analysis on pollinated *A. thaliana*, *A. lyrata* and *B. napus* stigmas. All pollinations were performed under low humidity conditions to ensure that the appropriate stigmatic responses to the pollen occurred [11], [42], [46], [47], [48], [49], [50], [51]. Our goal was to view secretory activity at the plasma membrane, and using fluorescently-tagged markers with live imaging was problematic as the stigmatic papillae have considerable background fluorescence that interferes with fluorescent signals. We were previously able to capture some data showing RFP-tagged Exo70A1 localized to the plasma membrane in *A. thaliana* stigmatic papillae; however, we were not able to visualize RFP:Exo70A1 in *B. napus* stigmatic papillae [17]. Thus, TEM was selected as the approach to view secretory activity at the plasma membrane in a comparative study across these three Brassicaceae species. Detailed TEM examinations of the pollen-papilla contact area were conducted at the selected time points, and the number of stigmatic papillae with secretory activity at the plasma membrane were scored.

**Figure 1:**
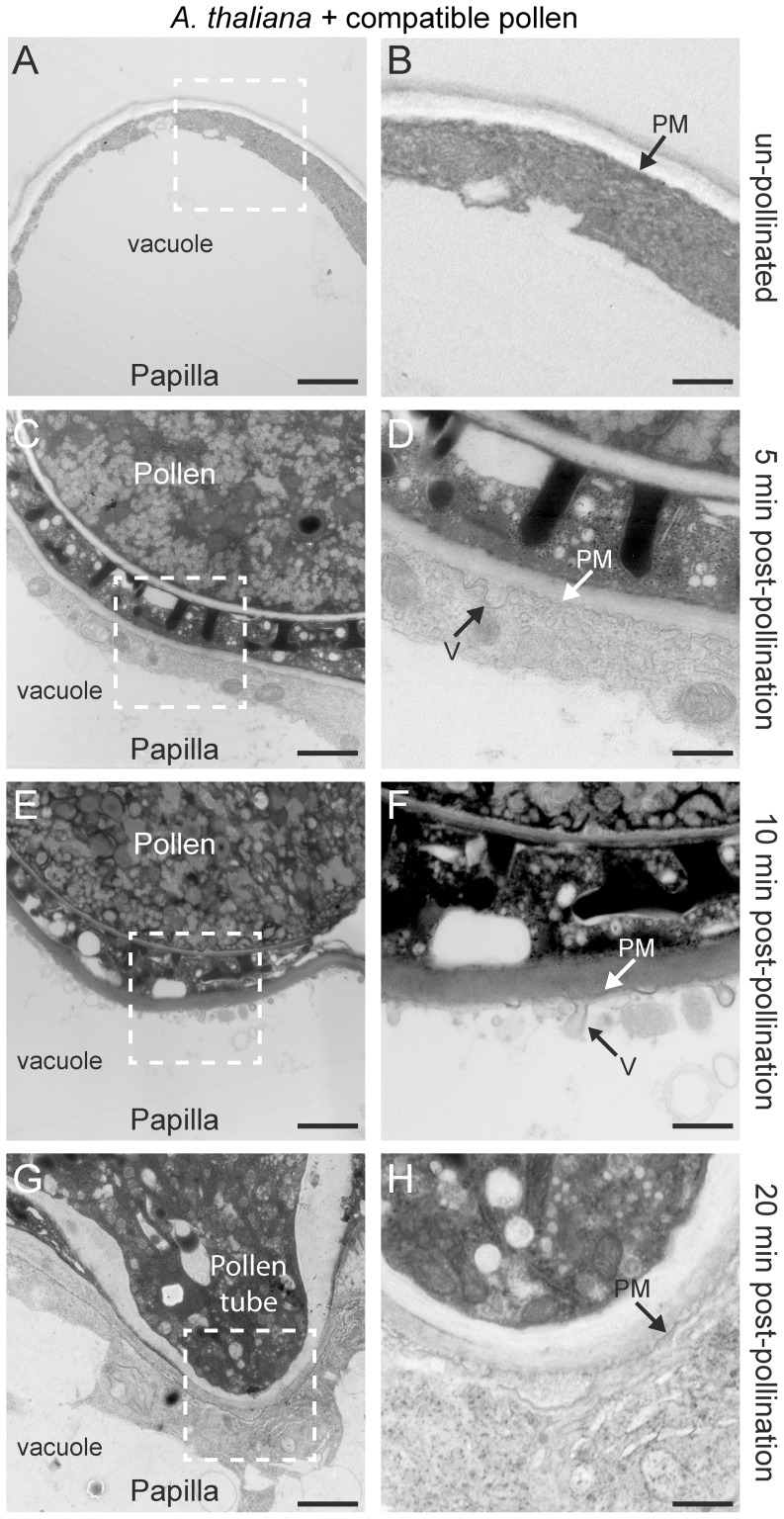
TEM images of *A. thaliana* Col-0 stigmatic papillae in response to self-compatible pollen. (**A, B**) Unpollinated stigmatic papilla. Secretory activity was not observed at the papillar plasma membrane (PM) in 10/10 samples. (**C, D**) Stigmatic papilla at 5 min post-pollination. Vesicles (V) were observed to be fusing to the plasma membrane (PM) underneath the pollen contact site in 25/25 samples. (**E, F**) Stigmatic papilla at 10 min post-pollination. Vesicles (V) continue to fuse to the plasma membrane (PM) underneath the pollen contact site in 25/25 samples. (**G, H**) Pollen tube penetration into the stigmatic papilla at 20 min post-pollination. Vesicles were no longer observed at the stigmatic papillar plasma membrane (PM) in 25/25 samples. The white boxed areas in (A, C, E, G) are shown in the (B, D, F, H), respectively. Scale bars (A, C, E, G) 1.5 µm; (B, D, F, H) 500 nm

**Figure 2:**
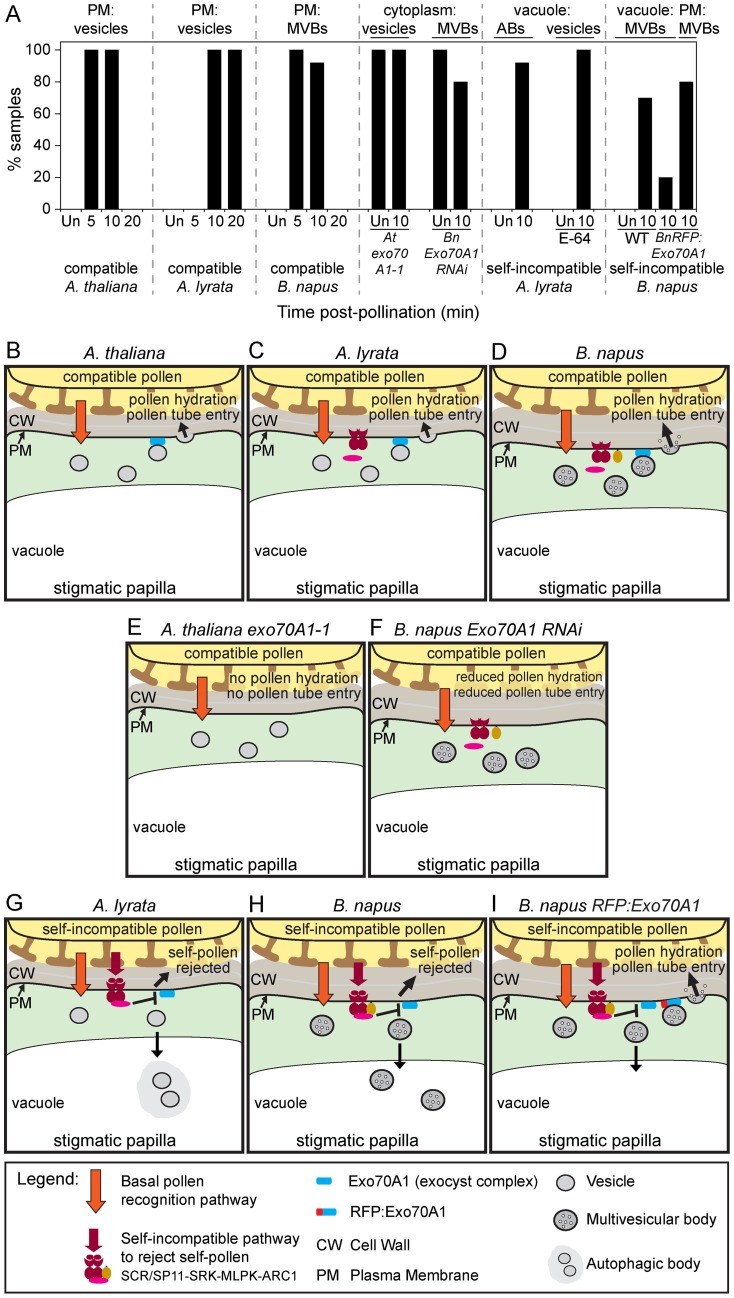
Summary of the stigmatic papillar responses to compatible and self-incompatible pollen. (**A**) Percentage of samples with the main ultrastructural features observed in the TEM images. Corresponding TEM images are shown in Figures 1, 4–7 and 9. Abbreviations: Un  =  unpollinated; MVBs  =  multivesicular bodies; ABs  =  autophagic bodies; PM  =  plasma membrane. (**B-D**) Models for compatible pollen responses (based on this study and cited references). Under the compatible pollen contact site, an unknown basal pollen recognition pathway is activated in the stigmatic papilla. This leads to the assembly of the exocyst complex with Exo70A1 and plasma membrane docking of vesicles in *Arabidopsis* species (B, C) or MVBs in *B. napus* (D). Following vesicle/MVB fusion to the plasma membrane by SNAREs, unknown cargo are released to facilitate water release for pollen hydration and cell wall expansion for pollen tube entry (pollen is accepted). (**E, F**) Models for loss of Exo70A1 function in the stigmatic papilla (based on this study and cited references). Disrupting *Exo70A1* expression prevents plasma membrane docking of vesicles in *A. thaliana exo70A1-1* (E) or MVBs in *B. napus Exo70A1* RNAi (F). Consequently, vesicles/MVBs accumulate in the cytoplasm; cargo needed for accepting the compatible pollen are not delivered to the plasma membrane; and this leads to pollen rejection. (**G, H**) Models for self-incompatible pollen responses (based on this study and cited references). With self-incompatible pollen, the self-incompatibility pathway is activated in the stigmatic papilla and overrides the basal pollen recognition pathway by inhibiting Exo70A1 and vesicle/MVB docking. The vesicles in *A. lyrata* (G) or MVBs in *B. napus* (H) are redirected to the vacuole via autophagy for degradation. Consequently, pollen hydration and pollen tube penetration are prevented (self-pollen is rejected). (**I**) Model for the partial breakdown of self-incompatibility through the expression of *RFP:Exo70A1* in *B. napus* [17]. MVBs are able to dock at the plasma membrane despite the activation of the self-incompatibility pathway, and this leads to pollen acceptance (pollen hydration and pollen tube entry occur [17]. RFP:Exo70A1 may potentially cause this phenotype through increased Exo70A1 levels or by interference with the ARC1 interaction.

In *A. thaliana*, vesicle-like structures were observed at the stigmatic papillar plasma membrane under the pollen grain at 5 minutes following a self-compatible pollination (Figure 1C, D; Figure 2A, B). The vesicles appeared to fuse with the plasma membrane and often contained an electron-opaque material, suggestive of cargo. The papillar plasma membrane in this area was quite ruffled in appearance (Figure 1D) in comparison to the unpollinated papilla (Figure 1B). This secretory activity appeared to be polarized towards the pollen grain as few vesicle-like structures could be observed at the flanking sides of the stigmatic papilla, and the plasma membrane was smoother in appearance. At ten minutes post-pollination, vesicles were again observed at the papillar plasma membrane underneath the pollen grain (Figure 1E, F). As previously reported [6], [39], the stigmatic papillar vacuolar network appeared to be oriented towards the pollen contact site, leading to a very thin layer of papillar cytoplasm at the pollen-papilla interface (Figure 1E, F). At 20 min, the pollen tube has emerged and is penetrating the stigmatic papillar outer wall [6], [58]. In contrast to the secretory activity observed at 5 and 10 min post-pollination, vesicle-like structures were not observed in the papillar cytoplasm under the pollen tube 20 min post pollination (Figure 1G, H). Pollen also has hydrolytic enzymes which may facilitate pollen tube penetration at the pollen-pistil interface [59], [60], [61], [62], and perhaps by 20 min, the pollen tube tip provides the necessary hydrolytic enzymes. As Exo70A1 was also found to be critical for the initial penetration of the pollen tube into the papillar surface [17], the secretory activity in the stigmatic papilla, prior to 20 min, is likely initiating this process.

**Figure 3:**
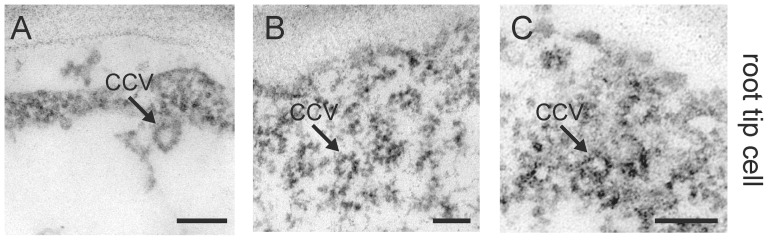
TEM images of clathrin-coated vesicles in the *A. thaliana* root tip cells. (**A-C**) The root tips from 6-day-old *A. thaliana* seedlings were observed as a reference for clathrin-coated vesicles (CCV) in the plant endocytic pathway. Clathrin-coated vesicles were observed adjacent to the plasma membrane in the root tip cells in 25/25 samples. Scale bars (A-C) 100 nm.

The timing of the appearance of vesicle-like structures in the stigmatic papilla in response to compatible pollen is consistent with these structures being secretory vesicles targeted to the compatible pollen contact site. The appearance of the ruffled plasma membrane is also consistent with active secretion occurring (and absent in the self-incompatible pollinations described below). While endocytosis typically accompanies secretion, it is unlikely to be the primary source of these vesicle-like structures. The major mechanism of endocytosis in plants is through clathrin-coated vesicles [63], and clathrin coats were not visible on these structures. To verify that this was not due to our experimental design, ultrastructural analysis was also performed on 6-day old Arabidopsis seedling root tips to visualize clathrin-coated vesicles [45]. On the basis of the typical morphology of clathrin-coated vesicles in root tips [45], we detected clathrin-coated vesicles (∼30 nm in diameter) at the internalization stage and before the loss of the clathrin coat in the root tip cells (Figure 3A-C). Such coated structures were not visible in the pollinated stigmatic papillae (Figure 1D, F).

**Figure 4:**
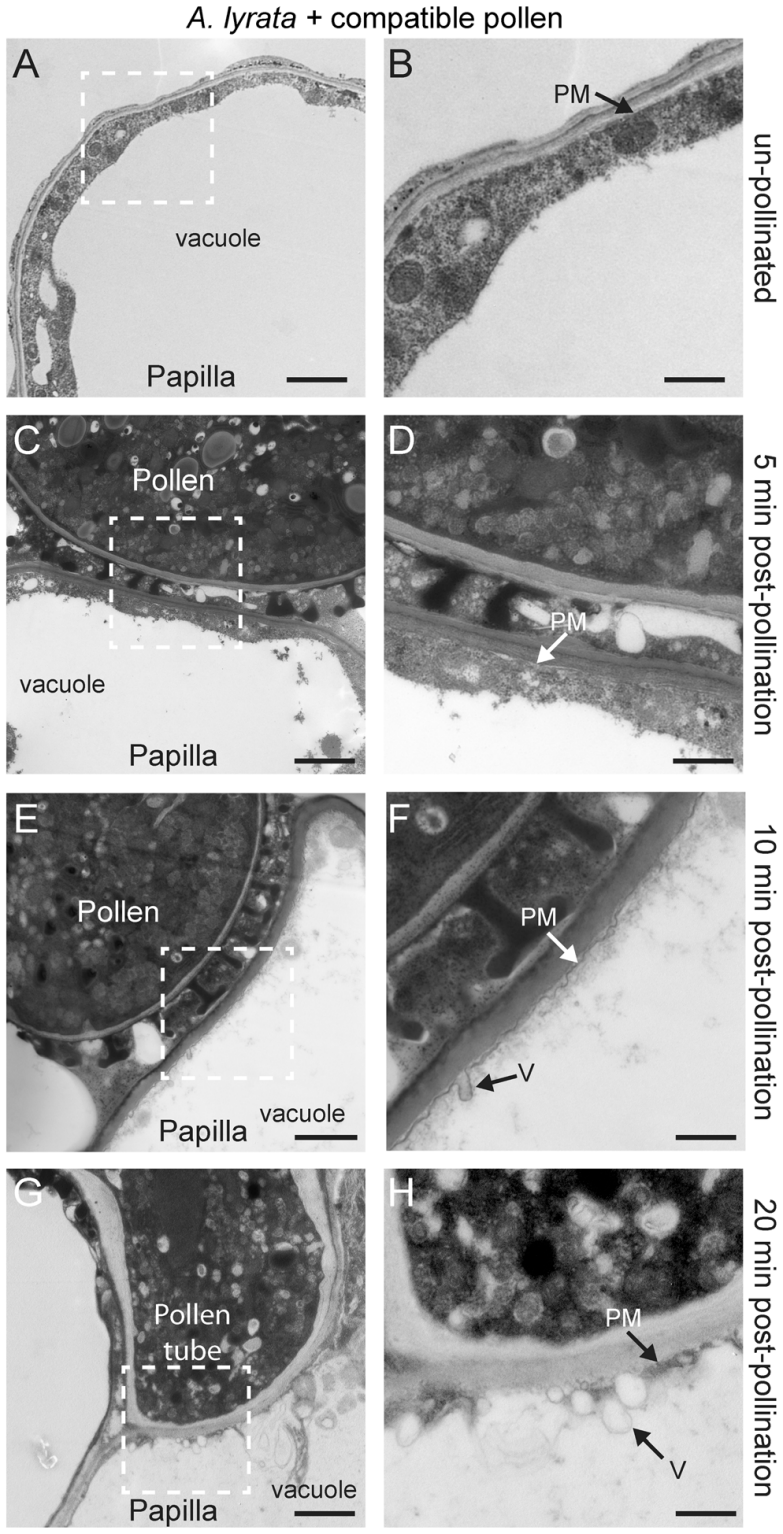
TEM images of *A. lyrata* stigmatic papillae in response to cross-compatible pollen. (**A, B**) Unpollinated stigmatic papilla. Secretory activity was not observed at the papillar plasma membrane (PM) in 10/10 samples. (**C, D**) Stigmatic papilla at 5 min post-pollination. Secretory activity was not observed at the papillar plasma membrane (PM) in 10/10 samples. (**E, F**) Stigmatic papilla at 10 min post-pollination. Vesicles (V) were observed to be fusing to the plasma membrane (PM) underneath the pollen contact site in 25/25 samples. (**G-H**) Pollen tube penetration into the stigmatic papilla at 20 min post-pollination. Vesicles (V) were observed at the papillar plasma membrane (PM) beneath the pollen tube tip in 25/25 samples. The white boxed areas in (A, C, E, G) are shown in the (B, D, F, H), respectively. Scale bars (A, C, E, G) 1.5 µm; (B, D, F, H) 500 nm.

The presence of vesicle secretion was also examined in another *Arabidopsis* species, self-incompatible *A. lyrata*, and compatible pollinations were conducted by using two cross-compatible plants (with different *S*-haplotypes; [24]). In general, secretory activity appeared to be delayed in *A. lyrata* by about 5 min when compared to *A. thaliana* (Figure 2A, C; Figure 4). Vesicle secretion appeared to be absent at 5 min post-pollination (Figure 4C, D); however, by 10 min after the cross-compatible pollination, vesicles appeared to fuse with the papillar plasma membrane under the pollen attachment site (Figure 4E, F). The papillar plasma membrane in this area was ruffled in appearance (Figure 4F) in contrast to the unpollinated papilla and at 5 min post-pollination (Figure 4B, D). The stigmatic papillar vacuolar network also appeared to be oriented towards the pollen contact site, resulting in a very thin layer of papillar cytoplasm (Figure 4E, F). Again in contrast to *A. thaliana*, secretory activity appeared to be continued in the papilla cell at the plasma membrane, 20 min post-pollination with the initial penetration of pollen tube (Figure 4G, H).

**Figure 5:**
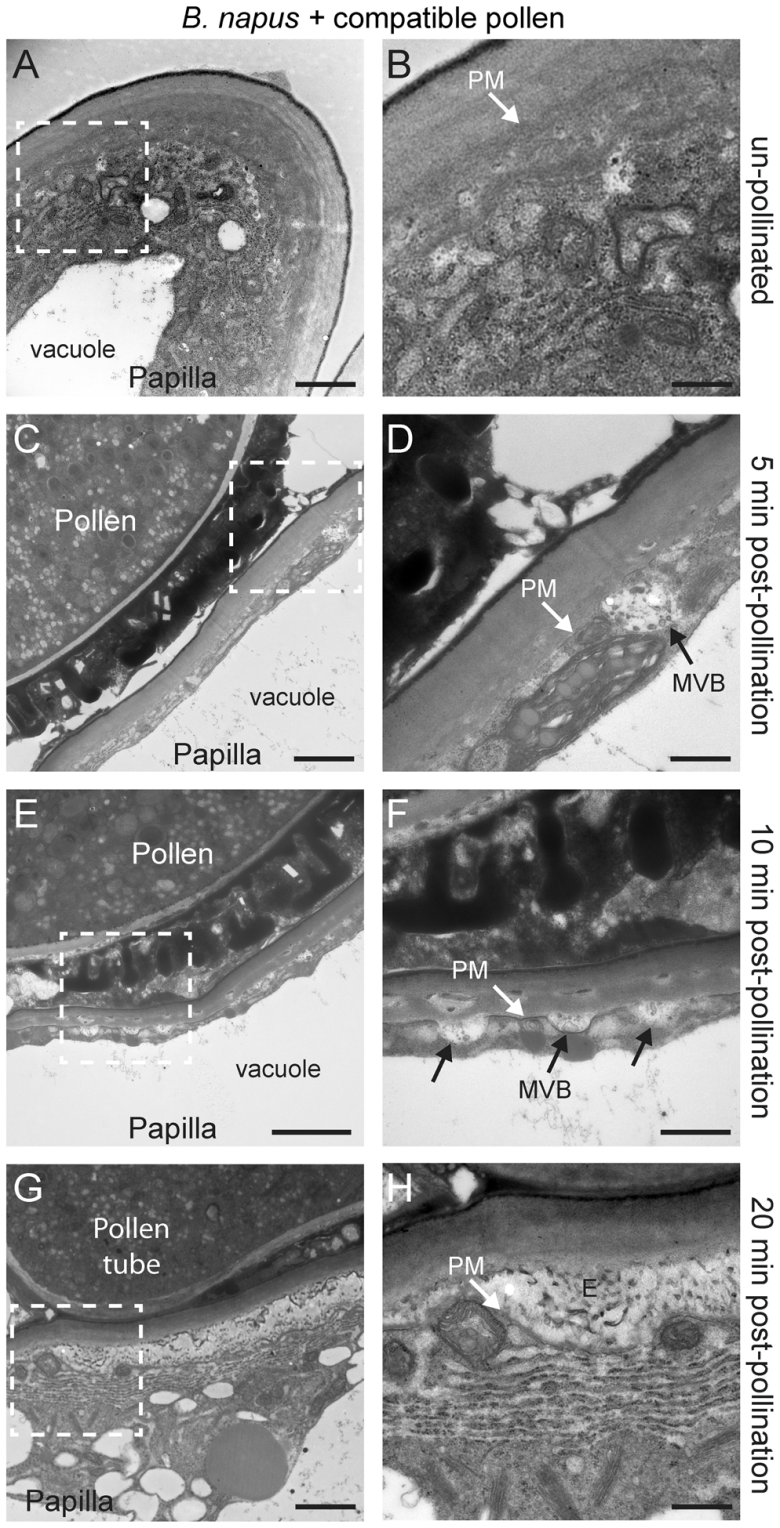
TEM images of *B. napus* Westar stigmatic papillae in response to self-compatible pollen. (**A, B**) Unpollinated stigmatic papilla. Secretory activity was not observed at the papillar plasma membrane (PM) in 10/10 samples. (**C, D**) Stigmatic papilla at 5 min post-pollination showing an MVB fusing to the plasma membrane (PM). MVBs at the plasma membrane were observed in 10/10 samples. (**E, F**) Stigmatic papilla at 10 min post-pollination showing several MVBs fusing to the plasma membrane (PM) underneath the pollen contact site. MVBs at the plasma membrane were observed in 23/25 samples. For 2/25 samples, vesicles were observed to be fusing to the plasma membrane (Figure S2A, B). (**G, H**) Pollen tube penetration into the stigmatic papilla at 20 min post-pollination. The material under the papillar cell wall appears to be the exosomes (E) released from the MVBs. This pattern was observed in 15/15 samples. The white boxed areas in (A, C, E, G) are shown in the (B, D, F, H), respectively. Scale bars (A, C, E, G) 1.5 µm; (B, D, F, H) 500 nm.

Finally, self-compatible pollinations in *B. napus* Westar cultivar were examined and interestingly, instead of individual secretory vesicles, multivesicular bodies (MVBs) appeared to be targeted to the stigmatic papillar plasma membrane under the pollen attachment (Figure 2A, D; Figure 5). The MVBs have multiple small vesicles surrounded by a membrane and were detected at the pollen contact site suggesting the existence of MVBs-mediated secretion pathway. In animals, MVBs have been found to secrete vesicles, termed exosomes, for intercellular communication [64], and MVBs have been proposed to secrete exosomes as part of defence responses to powdery mildew [65], [66]. MVBs ranged in size from 200 to 500 nm in diameter, and their intralumenal vesicles carried electron-opaque materials. At both 5 min and 10 min following the self-compatible pollination, MVBs were present at the papillar plasma membrane beneath the pollen contact site (Figure 5C-F). By 20 min, the pollen tube is just emerging and there appears to be a gap between the papillar cell wall and the plasma membrane filled with these secreted exosomes (Figure 5G, H). While *B. napus* has a larger stigma in comparison to the *Arabidopsis* species, the stigmatic papillae are similar in size (Figure S1) and so the switch to MVBs is not necessarily related to stigmatic papillar size. The *B. napus* stigmatic papillae did appear to have thicker cell walls compared to the *Arabidopsis* species (Figures 1, 4), and this change from individual secretory vesicles to MVBs may be related to a need for increased secretory activity across the cell wall to promote pollen hydration and pollen tube penetration. Nevertheless, secretory vesicles also appeared to be produced as 2/25 *B. napus* Westar papillae were observed to have vesicles fusing to the plasma membrane at 10 minutes post-pollination (Figure S2A, B).

### Impaired basal pollen recognition responses in stigmatic papillae from *A. thaliana* and *B. napus exo70A1* mutants

**Figure 6:**
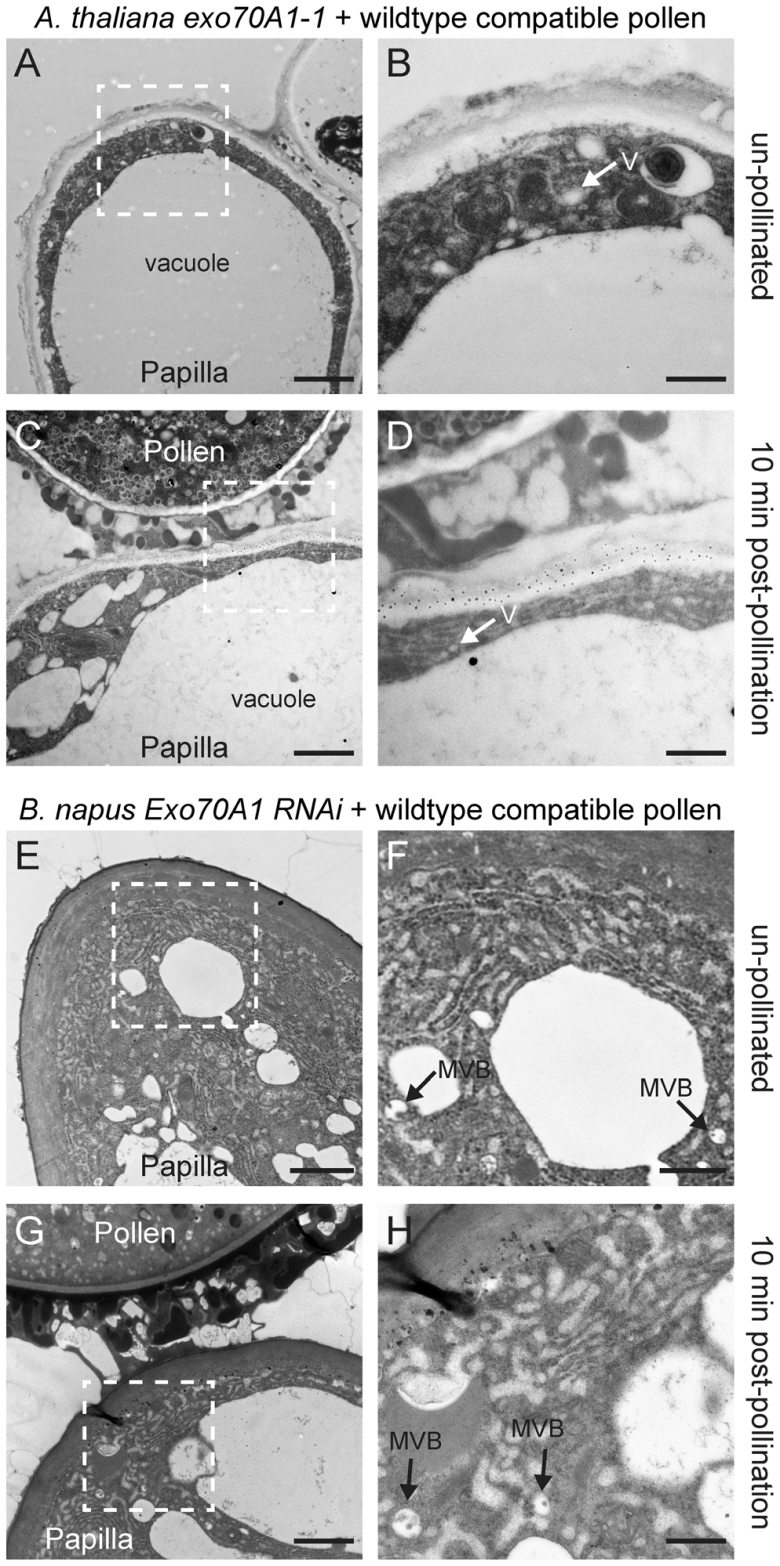
TEM images of *A. thaliana exo70A1-1* and *B. napus* Westar *Exo70A1* RNAi stigmatic papillae in response to wild-type compatible pollen. **(A, B)** Unpollinated stigmatic papilla from the *A. thaliana exo70A1-1* mutant. Some vesicle (V) accumulation was observed in the cytoplasm in 10/10 samples. **(C, D)** Stigmatic papilla from the *A. thaliana exo70A1-1* mutant at 10 min following pollination with compatible *A. thaliana* Col-0 pollen. An accumulation of secretory vesicles (V) in the papillar cytoplasm was observed under the pollen contact site in 10/10 samples. **(E, F)** Unpollinated stigmatic papilla from the *B. napus* Westar *Exo70A1* RNAi R2 line. Some accumulation of MVBs was observed in the cytoplasm in 10/10 samples. **(G, H)** Stigmatic papilla from the *B. napus* Westar *Exo70A1* RNAi R2 line at 10 min following pollination with compatible *B. napus* Westar pollen. Some MVBs were observed in the papillar cytoplasm in 8/10 samples. For 2/10 samples, MVBs were observed in the cytoplasm and fusing to the plasma membrane (Figure S2C, D) which is consistent with these plants displaying an incomplete knockout phenotype [17]. The white boxed areas in (A, C, E, G) are shown in the (B, D, F, H), respectively. Scale bars (A, C, E, G) 1.5 µm; (B, D, F, H) 500 nm.

In our previous work, while compatible *B. napus* and *A. thaliana* pollen grains were found to hydrate in the first 10 minutes following pollen application, wild-type pollen grains on either the *A. thaliana* Col-0 *exo70A1-1* mutant or the *B. napus* Westar *Exo70A1* RNAi stigmas showed very little increase in pollen diameter during this time indicating that pollen hydration was not occurring [17]. With this observation, we investigated whether vesicle/MVB secretion was impaired at 10 min following pollination with wild-type compatible pollen (Figure 2A, E, F; Figure 6). If disruption of Exo70A1 was correlated with a block in exocyst function, the typical phenotype that has been observed for exocyst mutants is an accumulation of secretory vesicles in the cytoplasm [30], [67], [68].

Pistils from the *A. thaliana* Col-0 *exo70A1-1* mutant were hand-pollinated with wild-type Col-0 pollen (compatible pollen) and examined at 10 min post-pollination. As expected, the *exo70A1-1* mutant stigmatic papilla had secretory vesicles accumulated in the cytoplasm (Figure 6C, D) which is comparable to the classical phenotype for yeast exocyst mutants [68]. In contrast to the pollinated wild-type stigmatic papilla, the vacuole in the stigmatic papilla did not appear to be oriented towards the pollen grain, the cytoplasm was not compressed, and vesicle fusion to the plasma membrane was not evident. Interestingly, the unpollinated *exo70A1-1* mutant stigmatic papilla also revealed some vesicle accumulation in the cytoplasm (Figure 6A, B). Similar observations were seen when stigmas from the *B. napus* Westar *Exo70A1* RNAi R2 line [17] were pollinated wild-type compatible *B. napus* Westar pollen. Some MVB accumulation in the cytoplasm was detected in both the unpollinated *B. napus* Westar *Exo70A1* RNAi stigmatic papillae (Figure 6E, F) and at 10 min post-pollination (Figure 6G, H). Again, the vacuole in the pollinated stigmatic papilla did not appear to be oriented towards the pollen grain, the cytoplasm was not compressed, and MVB fusion to the plasma membrane was not evident in most of the samples. In 2/10 samples, some MVB fusion at the plasma membrane was observed (Figure S2C, D), and this is in keeping with the *B. napus* Westar *Exo70A1* RNAi line being a knock-down line where some pollen adhesion and seed set were still observed [17]. Thus, the loss of Exo70A1 in both *A. thaliana* and *B. napus* inhibits the compatible pollen response and was correlated with the loss of vesicle/MVB fusion at the stigmatic papillar plasma membrane under the pollen contact site.

### Self-incompatible pollen responses in stigmatic papillae from *A. lyrata* and *B. napus*

**Figure 7:**
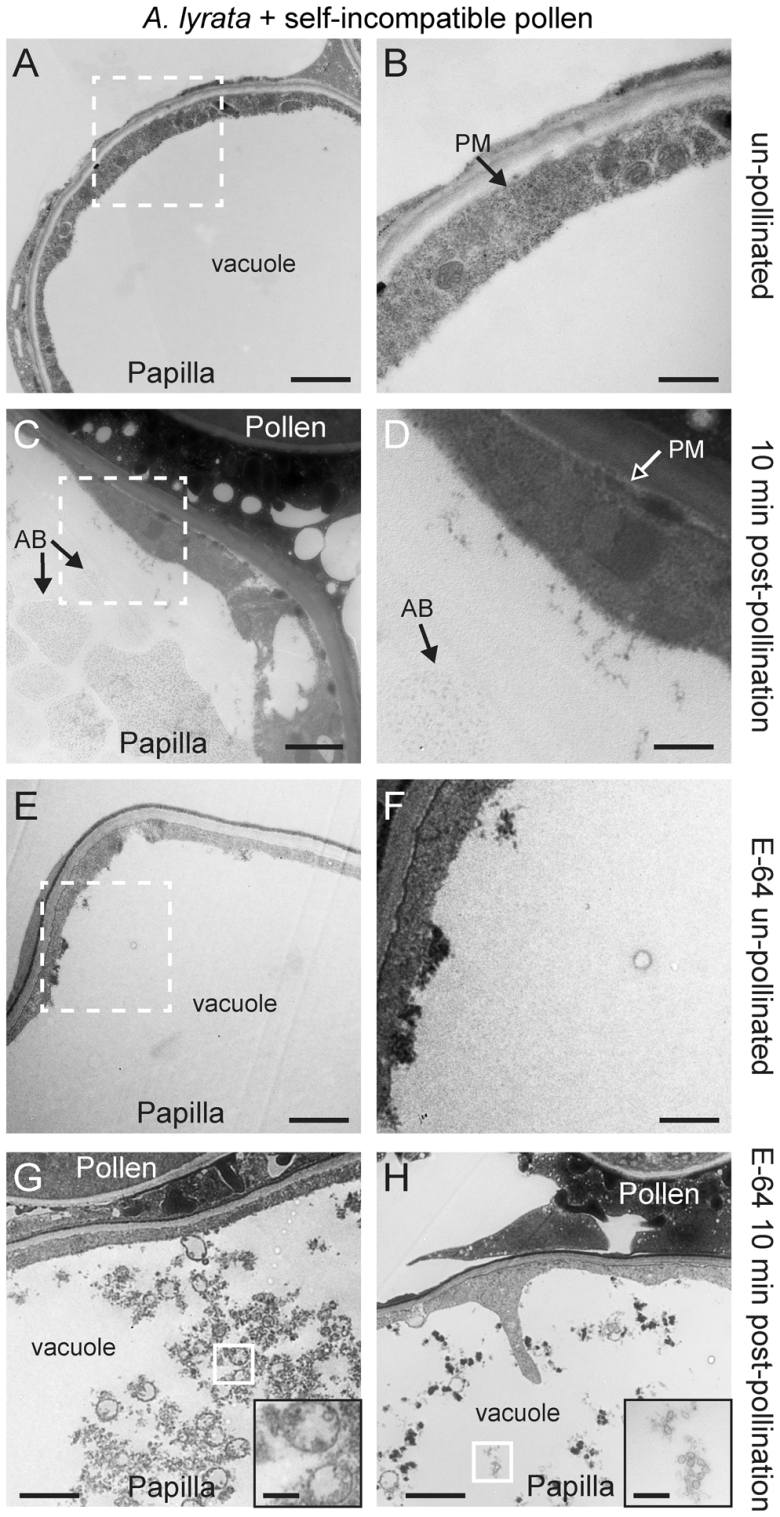
TEM images of *A. lyrata* stigmatic papillae in response to self-incompatible pollen. (**A, B**) Unpollinated stigmatic papilla. Secretory activity was not observed at the papillar plasma membrane (PM), and the vacuole was largely clear (i.e no autophagic bodies were visible) in 10/10 samples. (**C, D**) Stigmatic papilla at 10 min post-pollination with self-incompatible pollen. No secretory activity was observed at the papillar plasma membrane (PM). Structures that may represent autophagic bodies (AB) were observed in the vacuole in 23/25 samples. In 2/25 samples, these structures were not visible in the vacuole. (**E, F**) Unpollinated stigmatic papilla treated with the E-64 inhibitor. The vacuole was largely clear (i.e. no autophagic bodies or vesicles were visible) in 10/10 samples. (**G, H**) Stigmatic papillae treated with the E-64 inhibitor, at 10 min post-pollination with self-incompatible pollen. Un-degraded vesicles were observed in the vacuole in 10/10 samples. The white boxed areas in (A, C, E) are shown in the (B, D, F), respectively. The white boxed areas in (G) and (H) are shown in the insets in the bottom right hand corners. Scale bars (A, C, E, G, H) 1.5 µm; (B, D, F) 500 nm, Insets for (G, H) 300 nm.

With the model that the self-incompatibility response causes pollen rejection by inhibiting the basal pollen recognition responses in the stigmatic papillae, we predicted that there would also be a correlated loss of vesicle/MVB secretion at the plasma membrane. In line with this model, self-incompatible *B. napus* W1 pollen grains showed very little increase in pollen diameter at 10 min post-pollination indicating that pollen hydration was not occurring [17]. Thus, we investigated whether vesicle/MVB secretion was impaired at 10 min post-pollination for self-incompatible *A. lyrata* and the *B. napus* W1 cultivar (Figure 7).

At 10 min after a self-incompatible pollination in *A. lyrata* (Figure 7C, D), there was a complete absence of the features observed in the cross-compatible pollination (Figure 4E, F). Vesicles were absent from the stigmatic papillar plasma membrane underneath the self-incompatible pollen grain, the papillar plasma membrane was smooth in appearance (no ruffling), and the cytoplasm was not compressed by the vacuole (Figure 7C, D). This suggested that the vesicle secretion associated with the compatible pollen response was completely blocked in the self-incompatible pollen grain. Interestingly, in contrast to the *exo70A1-1* mutant, there was also an absence of vesicle accumulation in the papillar cytoplasm which suggested that the self-incompatible response went beyond simply inhibiting Exo70A1. Correlated with this was the appearance of dense material in the vacuole of 23/25 samples, and one possible explanation was that this material represented autophagic bodies. That is, autophagy was induced to direct the secretory vesicles to the vacuole for degradation. If this was the case, it would be difficult to observe secretory vesicles as only remnants of these structures would be discernible in the vacuole. The dense material accumulating in the vacuole was not observed in unpollinated papillae (Figure 7A, B).

To search for the accumulation of vesicles in the vacuole following a self-incompatible pollination, *A. lyrata* inflorescences were first treated with an inhibitor, E-64, for one day prior to pollination. E-64 is a cysteine protease inhibitor that blocks proteolysis in autophagic bodies and was previously shown to lead to the accumulation of undegraded cytoplasmic material in the central vacuole of Arabidopsis root tip cells [53]. Thus, we used the E-64 inhibitor treatment to see if undegraded vesicles could be observed in the stigmatic papillar vacuole. Unpollinated E-64 treated *A. lyrata* stigmatic papilla showed very little accumulation of cytoplasmic material in the vacuole (Figure 7E, F). However, at 10 min after a self-incompatible pollination, the *A. lyrata* stigmatic papillae accumulated cytoplasmic material in the vacuole, and within this material, vesicle-like structures could be seen (Figure 7G, H). Together, these results suggest that cellular components including vesicles are enclosed in autophagic bodies upon self-incompatibility, where they are transported into vacuoles and eventually degraded (Figure 2A, G).

**Figure 8:**
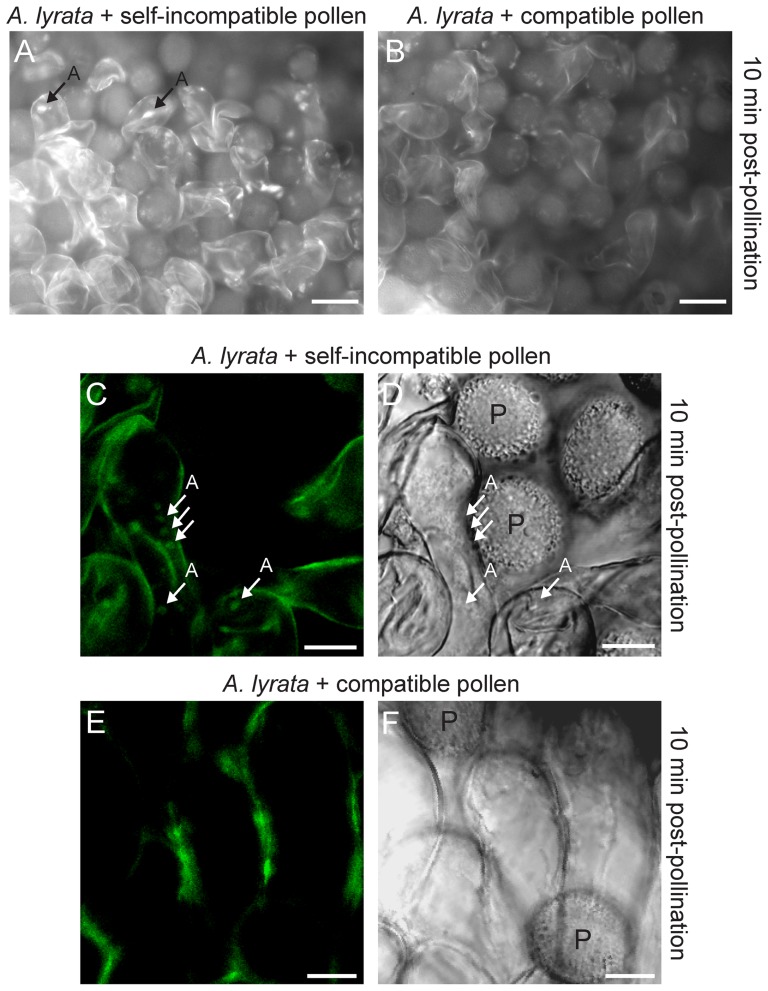
Autophagosomes in *A. lyrata* stigmatic papillae in response to self-incompatible pollen. (**A, B**) Florescence microscopy images of MDC stained *A. lyrata* stigmatic papillae at 10 min post-pollination. Fluorescent signals that may represent autophagosomes were seen in the *A. lyrata* stigmatic papillae following a self-incompatible pollination (A) in 10/10 samples, but not observed after a cross-compatible pollination (B) in 10/10 samples. (**C-F**) Confocal microscopy images of transgenic *A. lyrata* GFP:ATG8a stigmatic papillae at 10 min post-pollination. GFP:ATG8a is a marker for autophagy induction, and GFP signals marking potential autophagosomes were observed in the stigmatic papillae following a self-incompatible pollination (C) in 10/10 samples (corresponding DIC image is shown in D). Punctate GFP signals were not detected within the stigmatic papillae following a cross-compatible pollination (E) in 10/10 samples (corresponding DIC image is shown in F). All samples, including wild-type untransformed *A. lyrata* stigmatic papillae showed background fluorescence from the cell wall. A  =  autophagosomes; P =  pollen. Scale bars (A, B) 50 µm; (C-F) 10 µm.

Two other approaches were used to assess whether autophagy was induced as part of the self-incompatibility response in *A. lyrata*: staining of pollinated stigmas with the fluorescent dye, monodansylcadaverine (MDC), and generating transgenic *A. lyrata* expressing a Green Fluorescent Protein (GFP)-ATG8a fusion protein. The MDC dye accumulates in the lipid-rich membranes that are highly concentrated in the autophagic compartments and thus, is used for *in vivo* labeling of autophagosomes in cells [55], [69]. *Arabidopsis* ATG8a is an ubiquitin-like protein that is attached to the membrane during autophagosome formation, and thus, marks autophagosomes destined for the vacuole [56]. Cross-compatible pollinations were performed as a control for the absence of autophagy. At 10 min post self-incompatible pollination, MDC-labeled structures appeared to accumulate in the stigmatic papillae (Figure 8A) when examined under the fluorescence microscopy. Fluorescent signals resembling MDC-labeled autophagosomes were not detected in cross-compatible pollinated stigmatic papillae (Figure 8B). The potential induction of autophagy in self-incompatible pollinated stigmatic papillae was further examined by transforming the autophagy marker, GFP:ATG8a, into *A. lyrata* plants, and performing both self-incompatible and cross-compatible pollinations. Using confocal microscopy, all stigma samples, including untransformed stigmatic papillae, gave some background fluorescence in the cell walls (Figure 8E). However, specific GFP fluorescence from GFP:ATG8a-labelled intracellular structures were observed in *A. lyrata* papillae following self-incompatible pollination (Figure 8C). These structures were not detected in the cross-compatible pollinated stigmatic papillae (Figure 8E). Thus, both the MDC staining and GFP:ATG8a marker suggest that autophagosomes are accumulating in the stigmatic papillae pollinated with self-incompatible pollen, and that autophagy may be responsible for clearing secretory vesicles from the papillae as part of the self-pollen rejection response.

**Figure 9:**
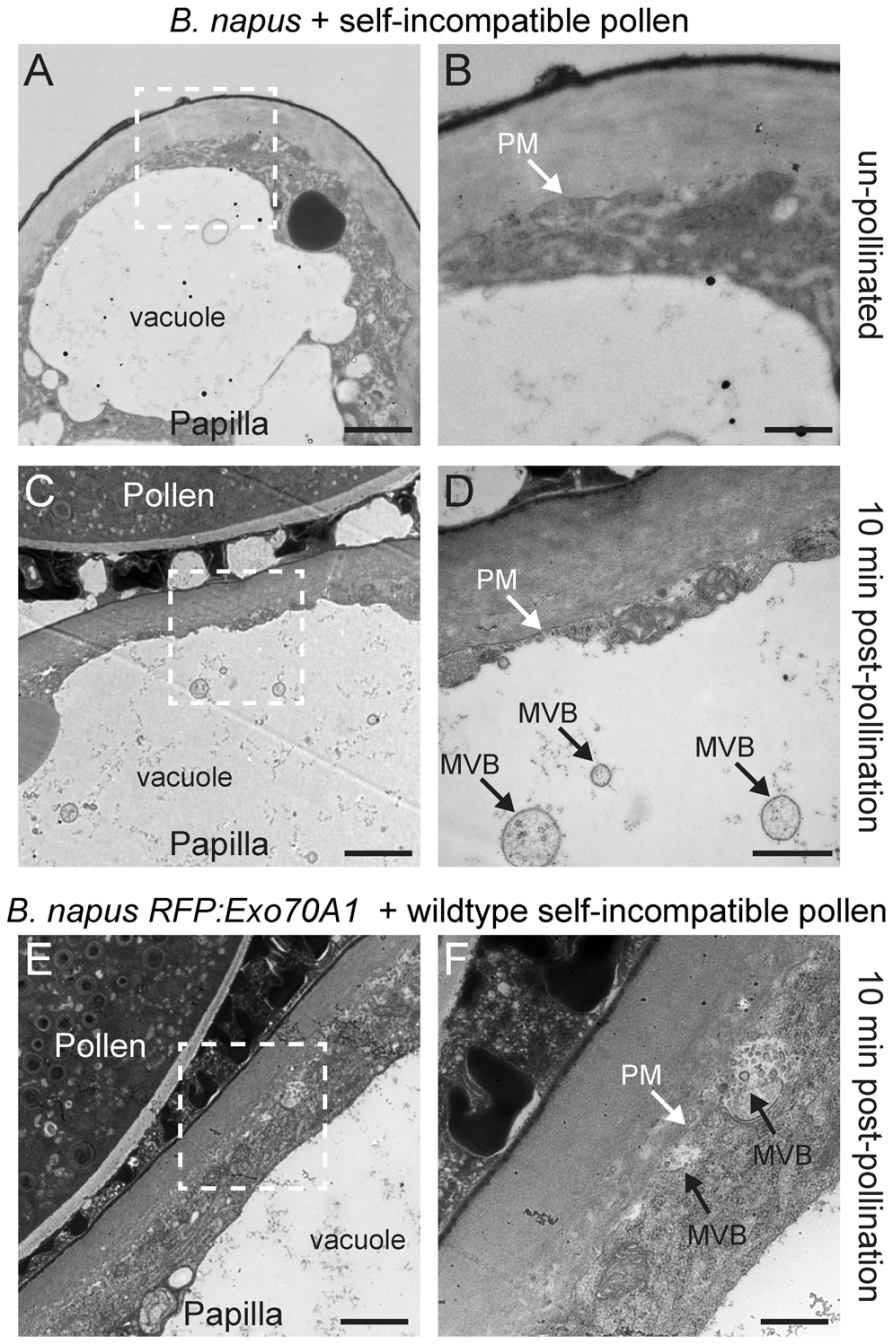
TEM images of *B. napus* W1 stigmatic papillae in response to self-incompatible pollen. (**A, B**) Unpollinated *B. napus* W1 stigmatic papilla. Secretory activity was not observed at the papillar plasma membrane (PM), and the vacuole was largely clear (no MVBs were visible) in 10/10 samples. (**C, D**) *B. napus* W1 stigmatic papilla at 10 min post-pollination with self-incompatible pollen. Secretory activity was not observed at the papillar plasma membrane (PM). Instead, MVBs were observed in the vacuole in 7/10 samples. For 3/10 samples, MVBs were observed in the cytoplasm near the vacuole (Figure S2E, F). (**E, F**) Transgenic *B. napus RFP:Exo70A1* W1 stigmatic papilla at 10 min post-pollination with self-incompatible pollen. These plants were previously found to have a partial breakdown of the self-incompatibility response due to *RFP:Exo70A1* expression [17]. Consistent with this partial phenotype, MVBs were observed to be fusing to the plasma membrane in 8/10 samples as shown in (F), and MVBs were also observed in the vacuole in 2/10 samples (Figure S2G, H). The white boxed areas in (A, C, E) are shown in the (B, D, F), respectively. Scale bars (A, C, E) 1.5 µm; (B, D, F) 500 nm.

At 10 min following a self-incompatible pollination with the *B. napus* W1 cultivar (Figure 9C, D), there was again a complete absence of the features observed in the self-compatible pollination with the *B. napus* Westar cultivar (Figure 5E, F). Most notably, there was an absence of MVBs fusing with the stigmatic papillar plasma membrane underneath the self-incompatible pollen grain. Interestingly in 7/10 samples, MVBs were now present in the vacuole (Figure 9C, D), and this suggests that the self-pollen rejection response targets MVBs to the vacuole for degradation in the stigmatic papilla (Figure 2A, H). In 3/10 samples, MVBs were present in the cytoplasm, near the vacuole. The accumulation of MVBs in the vacuole was not observed in unpollinated papillae (Figure 9A, B). In our previous work, the expression of an *RFP:Exo70A1* fusion in the stigmas of self-incompatible W1 plants was found to partially overcome self-incompatibility [17]. To see what effect *RFP:Exo70A1* had on the MVB distribution, transgenic *RFP:Exo70A1* W1 lines were examined at 10 min post-pollination. Mixed results were seen which would occur with the incomplete self-incompatibility phenotype observed in these lines (Figure 2A, I). For 8/10 samples, MVBs were observed to be fusing to the papillar plasma membrane under the pollen contact site following self-pollination (Figure 9E, F), though they were not as abundant as that observed for a fully compatible pollination (Figure 5E, F). For 2/10 samples, MVBs were found in the vacuole which would be representative of a self-incompatibility response. Thus, in response to pollen, MVBs in the stigmatic papilla may be sorted to distinct pathways based on whether the pollen is accepted or rejected (Figure 2A, D, H).

## Discussion

Upon pollen landing on the Brassicaceae stigmatic papillae, cell-cell communication events trigger distinct cascades in the early stages leading to successful compatible pollen acceptance or self-incompatible pollen rejection. This ensures that a plant's resources are reserved solely for the most appropriate pollen grains for fertilization and seed set [70]. Our previous work has implicated Exo70A1 as the protein that is regulated in the basal pollen recognition pathway and the self-incompatible pollen rejection pathway [17]. Exo70 is a subunit of the exocyst, a complex composed of the Sec3, Sec5, Sec6, Sec8, Sec10, Sec15, Exo70 and Exo84 subunits. In plants, the Exo70 gene has undergone a large gene expansion, and Exo70A1 represents one member of this family [28], [71]. In yeast and animal systems, the function of the exocyst is to assemble and dock secretory vesicles at specific sites at the plasma membrane where polar secretion is required (reviewed in [72], [73]). Yeast Exo70 subunit has been shown to present at the plasma membrane prior to exocyst assembly and vesicle tethering [74], and we have similarly seen *Arabidopsis* Exo70A1 localized to the stigmatic papillar plasma membrane prior to pollination [17]. Thus, in our working model, a compatible pollination leads to an unknown signal activating the basal pollen recognition pathway in the stigmatic papilla to assemble the exocyst complex at the plasma membrane underneath the pollen grain for polar secretion (see models in Figure 2B-D). With the interaction of Exo70A1 with the ARC1 self-incompatibility factor and the pollination phenotypes of *exo70A1* mutant plants, we proposed that self-incompatible pollen rejection occurs by overriding the basal pollen recognition pathway through the inhibition Exo70A1 and blocking the exocyst mediated polar secretion [17] (see models in Figure 2G, H). In this study, we investigated one prediction of this model: whether vesicle secretion was detected with compatible pollinations and absent in self-incompatible pollinations.

Our results for compatible pollinations in *A. thaliana* and *A. lyrata* support that vesicular transport occurs at the stigmatic papillar plasma membrane beneath the compatible pollen grain. The timing of the appearance of these vesicle-like structures was dynamic with vesicles detected at 5 and 10 min post-pollination for *A. thaliana*, and 10 and 20 min post-pollination for *A. lyrata*. This correlates well with the timing of pollen hydration, one of the processes regulated by the stigmatic papilla in the compatible pollen response [9], [10], [11]. These vesicle-like structures are specific to compatible pollinations as they were not detected at the stigmatic papillar plasma membrane with *A. lyrata* self-incompatible pollinations or when the *Exo70A1* gene was disrupted (*A. thaliana exo70A1-1* mutant). However, one question that arises is whether these vesicles are fusing to (exocytosis) or budding off (endocytosis) from the papillar plasma membrane under the pollen attachment site. While animal cells make use of multiple endocytic pathways, including clathrin-dependent, caveolin-dependent, or clathrin/caveolin-independent pathways [75], clathrin-mediated endocytosis is the main pathway of endocytosis in plant cells [63]. We were able to observe clathrin-coated endocytic vesicles in *Arabidopsis* seedling root tips as previously published [45]; however, clathrin-coated vesicles were not observed with compatible pollinations. Nevertheless, dynamic vesicle trafficking typically includes both exocytosis and endocytosis to maintain plasma membrane integrity and so one would expect some endocytosis to be simultaneously occurring. The stigmatic papillar membrane ruffling observed following compatible pollinations in *A. thaliana* and *A. lyrata* may indicate that endocytosis is occurring at a slower rate than the very active exocytosis towards the compatible pollen grain.

A surprising finding in our study was the observation of putative MVBs fusing with the papillar plasma membrane at 5 and 10 min following compatible pollinations in *B. napus* Westar cultivar. Similar to the *Arabidopsis* secretory vesicles, MVBs at the papillar plasma membrane were specific to compatible pollinations, being absent with self-incompatible pollinated *B. napus* W1 papillae and in the *B. napus* Westar *Exo70A1* RNAi papillae. They were also observed at the papillar plasma membrane of self-pollinated transgenic *B. napus RFP:Exo70A1* W1 stigmas (these lines had been previously found to have a compromised self-incompatibility response; [17]). Although MVBs typically function in recycling and endocytosis, they have also been identified in cellular processes that rely on polarized secretion and exocytosis [64], [76]. In plants, such processes include pathogen encasement and callose deposition during a defense response to powdery mildew fungal infection [65], [66], [76], [77], [78]. With *B. napus* compatible pollinations, the fusion of the MVBs with the papillar plasma membrane would result in the release of the internal vesicles (exosomes) into the apoplastic space. By 20 min post-compatible pollination, a buildup of exosome-like structures could be observed between the papillar plasma membrane and the cell wall under the pollen grain. Interestingly, the release of exosomes does offer an explanation for the previously published work by Elleman & Dickinson [37] where they treated *B. oleracea* stigmatic papillae for 20 min with coating extracted from compatible pollen, and observed vesicle-like structures within the expanded papillar cell wall. Elleman & Dickinson [37] also observed that these vesicle-like structures appeared to be fusing under the papillar cuticle to release their contents.

For both the self-incompatible pollinations using *A. lyrata* or *B. napus* W1, the vesicles/MVBs were absent from the stigmatic papillar membrane under the pollen grain. From our model, we had predicted that the self-incompatibility pathway would block the basal pollen recognition pathway through ARC1's inhibition of Exo70A1 would prevent vesicle/MVB secretion at the papillar plasma membrane (Figure 2G, H). One possible outcome from this would be that the self-incompatible pollinated stigmatic papilla would appear like an exocyst mutant with the accumulation of vesicles/MVBs in the cytoplasm (Figure 2E, F). For example, this was observed for the pollinated *A. thaliana exo70A1* and *B. napus* Westar *Exo70A1* RNAi stigmatic papillae. However, the self-incompatibility response in both *A. lyrata* and *B. napus* W1 appeared to go one step further and target vesicles/MVBs to the vacuole for degradation (Figure 2G, H). This was clearly seen for the *B. napus* W1 stigmatic papillae where the MVBs were found inside the vacuole at 10 min post-self-incompatible pollination. For *A. lyrata*, vesicles could not be observed in the vacuole; however, dense material was found to accumulate in the vacuole at 10 min post-self-incompatible pollination. Pre-treatment with the E-64 cysteine protease inhibitor, which inhibits the proteolytic activity in autophagic bodies, did allow for the detection of vesicle-like structures in the vacuole, following a self-incompatible pollination. It has been previously shown that when a constitutive autophagy process is blocked in *Arabidopsis* root tip cells by E-64 treatment, there is an accumulation of undegraded cytoplasmic material in the central vacuole [53]. Since unpollinated stigmatic papillae pre-treated with E-64 did not show the same accumulation of cytoplasmic material and vesicle-like structures in the vacuole, autophagy appeared to be induced in *A. lyrata* stigmatic papillae following self-incompatible pollinations. This was verified using two different autophagosome markers, MDC and GFP-ATG8a.

As with other eukaryotic cells, autophagy is one of the major pathways for degradation of intracellular macromolecules in plant cells [55], [56] Autophagy can be induced with environmental stress conditions or during certain stages of development, and upon induction, targeted cytoplasmic components are enclosed by membrane sacs to produce a double-membrane bound autophagosome. The autophagosomes are transported into the vacuole to degrade the sequestered materials (reviewed in [79], [80]). The induction of autophagy following a self-incompatible pollination would help in pollen rejection by clearing vesicles/MVBs from the cytoplasm. It may also help to explain why we previously observed the down-regulation of a number of proteins from metabolic pathways and organelles following self-incompatible pollinations [41]. Perhaps autophagy also contributes to nutrient recycling so that the plant's nutrients are reserved solely for those pollen grains most likely to lead to fertilization and seed set. Interestingly, one of the exocyst subunits, Exo84, has been found in mammalian cells to play a role in autophagosome formation during nutrient starvation and pathogen responses [73], [81]. The *Arabidopsis* Exo70B1 subunit has also been recently implicated in autophagy [82]. Whether a dual role in vesicle secretion and autophagy for specific exocyst subunits exists during pollen-pistil interactions is yet to be determined.

In conclusion, this study presents data that supports the model for the induction of exocytosis in stigmatic papillae as part of the basal pollen recognition pathway in response to compatible pollen and the requirement of Exo70A1 for this process (Figure 2B-D, E, F, I). While vesicles were observed in the *Arabidopsis* species, MVBs secreting exosomes were observed in *B. napus*. The switch from secretory vesicles in the *Arabidopsis* species to MVBs in *B. napus* may be related to the increased thickness of the papillar cell wall in *B. napus* (Figures 1, 4, 5) as all three species had similar sized stigmatic papillae (Figure S1). The cargo of the secretory vesicles is unknown, but possible candidates include aquaporins for water transport to the pollen grain. For example, aquaporins have been identified in secretory vesicle membranes, targeted to the plasma membrane via the exocyst complex, to increase water permeability in renal duct cells [83]. The vesicles may also contain hydrolytic enzymes to loosen the papillar cell surface for pollen tube penetration. Furthermore, the data in this study supports the model for the inhibition of exocytosis in the stigmatic papilla in response to self-incompatible pollen (Figure 2G, H). The early stigmatic responses to compatible and self-incompatible pollen are very rapid, and self-incompatible pollen would initially activated both pathways. That is, the basal pollen recognition pathway leading to vesicle/MVB secretion is initiated, and then the self-incompatibility pathway must activate a mechanism to block secretion to reject the self-incompatible pollen (Figure 2G, H). Our data suggests that the inhibition of the exocyst through Exo70A1 degradation is one step to prevent vesicle/MVB from docking at the stigmatic papillar plasma membrane under the pollen contact site (this study, [17]). The second step is the targeting of vesicles/MVBs to the vacuole for degradation possibly through autophagy. Iwano et al. [39] also observed that actin filaments were depolymerized, which would disrupt vesicle trafficking, and that the vacuolar network, a possible source of water for pollen hydration, appeared to be more fragmented. Future studies will need to address how all these different events are connected to the SRK-activated self-incompatibility response.

## Supporting Information

Figure S1 **Flower and stigma sizes are diversified in the Brassicaceae.** Images of (**A**) *B. napus* and *A. thaliana* flowers, (**B**) *A. lyrata* flower, (**C**) *A. lyrata* and *A. thaliana* stigmas, and (**D**) *B. napus* and *A. thaliana* stigmas. *B. napus* possesses a larger flower and stigma size compared to *A. lyrata* and *A. thaliana*. This increased size may result in different patterns of secretory activity to promote pollen acceptance (i.e. multivesicular bodies versus vesicles). However, there are no obvious differences in the sizes of the stigmatic papillae. Both *B. napus* and *A. lyrata* stigmas are densely packed with stigmatic papillae in comparison to the *A. thaliana* stigma. Scale bars (A, B) 1 mm; (C, D) 0.5 mm.
(PDF)

Figure S2 **TEM images of B. napus stigmatic papillae showing alternate trafficking patterns.** (**A, B**) B. napus Westar stigmatic papilla at 10 min post-pollination with self-compatible pollen. Vesicles (V) appear to be fusing to the papillar plasma membrane (PM) underneath the pollen contact site in 2/25 samples. (**C, D**) B. napus Westar Exo70A1 RNAi stigmatic papilla at 10 min post-pollination with wild-type compatible pollen. MVBs appear to fuse with the plasma membrane (PM) in 2/10 samples. This observation is consistent with the incomplete knockout phenotype of the B. napus Westar Exo70A1 RNAi plants [17]. (**E, F**) B. napus W1 stigmatic papilla at 10 min post-pollination with self-incompatible pollen. MVBs appear to be targeted to the vacuole after self-incompatible pollinations. These images show a potential fusion of a MVB to the vacuole, and intact MVBs were not visible inside the vacuole as observed in 3/10 samples. (**G, H**) B. napus RFP:Exo70A1 W1 stigmatic papilla at 10 min post-pollination with self-incompatible pollen. MVBs were observed in the vacuole in 2/10 samples which is consistent with these plants displaying an incomplete self-incompatibility response [17]. The white boxed areas in (A, C, E, G) are shown in the (B, D, F, H), respectively. Scale bars (A, C, E, G) 1.5 µm; (B, D, F, H) 500 nm.
(PDF)
