# Supplementary material for: Secretory Activity Is Rapidly Induced in Stigmatic Papillae by Compatible Pollen, but Inhibited for Self-Incompatible Pollen in the Brassicaceae
Source: PLoS One. 2013 Dec 26;8(12):e84286. doi: 10.1371/journal.pone.0084286 (PMC3873414; doi:10.1371/journal.pone.0084286)
Supplement: Figure S1 — Flower and stigma sizes are diversified in the Brassicaceae. Images of (A) B. napus and A. thaliana flowers, (B) A. lyrata flower, (C) A. lyrata and A. thaliana stigmas, and (D) B. napus and A. thaliana stigmas. B. napus possesses a larger flower and stigma size compared to A. lyrata and A. thaliana. This increased size may result in different patterns of secretory activity to promote pollen acceptance (i.e. multivesicular bodies versus vesicles). However, there are no obvious differences in the sizes of the stigmatic papillae. Both B. napus and A. lyrata stigmas are densely packed with stigmatic papillae in comparison to the A. thaliana stigma. Scale bars (A, B) 1 mm; (C, D) 0.5 mm. (PDF) [file pone.0084286.s001.pdf]

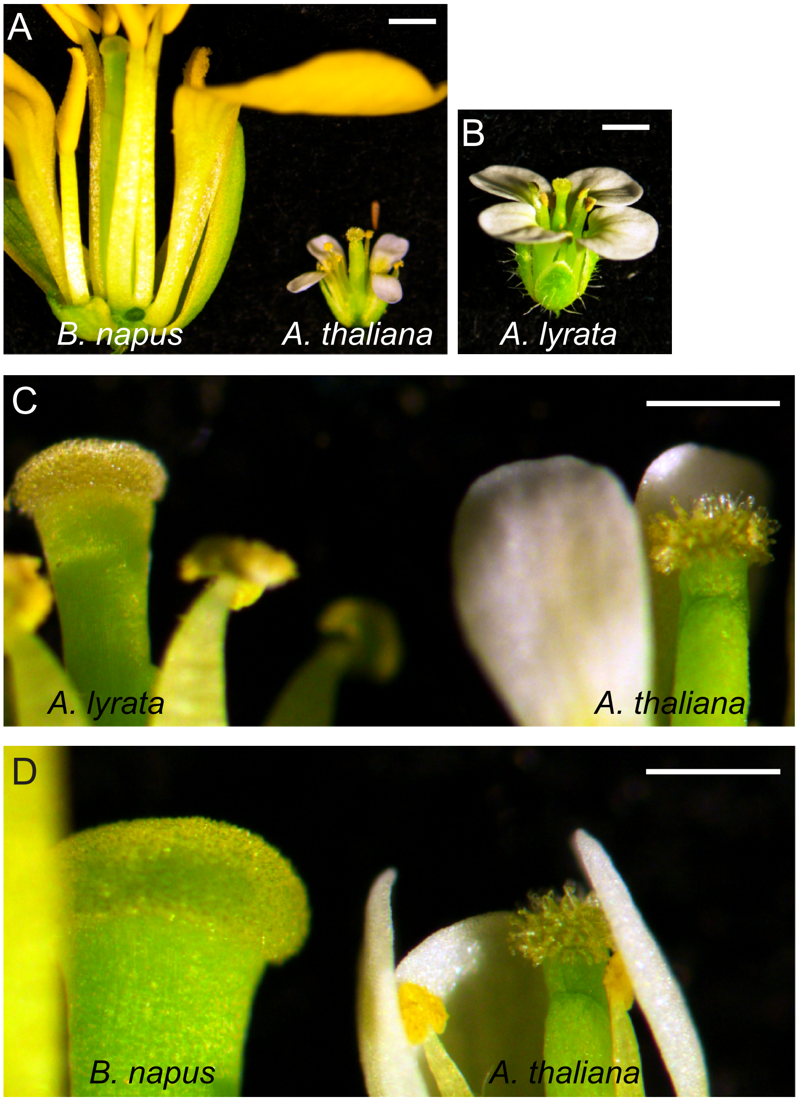


**Figure S1. Flower and stigma sizes are diversified in the Brassicaceae.** Images of **(A)** *B. napus* and *A. thaliana* flowers, **(B)** *A. lyrata* flower, **(C)** *A. lyrata* and *A. thaliana* stigmas, and **(D)** *B. napus* and A. *thaliana* stigmas. *B. napus* possesses a larger flower and stigma size compared to *A. lyrata* and *A. thaliana*. This increased size may result in different patterns of secretory activity to promote pollen acceptance (i.e. multivesicular bodies versus vesicles). However, there are no obvious differences in the sizes of the stigmatic papillae. Both *B. napus* and *A. lyrata* stigmas are densely packed with stigmatic papillae in comparison to the *A. thaliana* stigma.
Scale bars (a, b) 1 mm; (c, d) 0.5 mm.
