# Supplementary material for: Secretory Activity Is Rapidly Induced in Stigmatic Papillae by Compatible Pollen, but Inhibited for Self-Incompatible Pollen in the Brassicaceae
Source: PLoS One. 2013 Dec 26;8(12):e84286. doi: 10.1371/journal.pone.0084286 (PMC3873414; doi:10.1371/journal.pone.0084286)
Supplement: Figure S2 — TEM images of B. napus stigmatic papillae showing alternate trafficking patterns. (A, B) B. napus Westar stigmatic papilla at 10 min post-pollination with self-compatible pollen. Vesicles (V) appear to be fusing to the papillar plasma membrane (PM) underneath the pollen contact site in 2/25 samples. (C, D) B. napus Westar Exo70A1 RNAi stigmatic papilla at 10 min post-pollination with wild-type compatible pollen. MVBs appear to fuse with the plasma membrane (PM) in 2/10 samples. This observation is consistent with the incomplete knockout phenotype of the B. napus Westar Exo70A1 RNAi plants [17]. (E, F) B. napus W1 stigmatic papilla at 10 min post-pollination with self-incompatible pollen. MVBs appear to be targeted to the vacuole after self-incompatible pollinations. These images show a potential fusion of a MVB to the vacuole, and intact MVBs were not visible inside the vacuole as observed in 3/10 samples. (G, H) B. napus RFP:Exo70A1 W1 stigmatic papilla at 10 min post-pollination with self-incompatible pollen. MVBs were observed in the vacuole in 2/10 samples which is consistent with these plants displaying an incomplete self-incompatibility response [17]. The white boxed areas in (A, C, E, G) are shown in the (B, D, F, H), respectively. Scale bars (A, C, E, G) 1.5 µm; (B, D, F, H) 500 nm. (PDF) [file pone.0084286.s002.pdf]

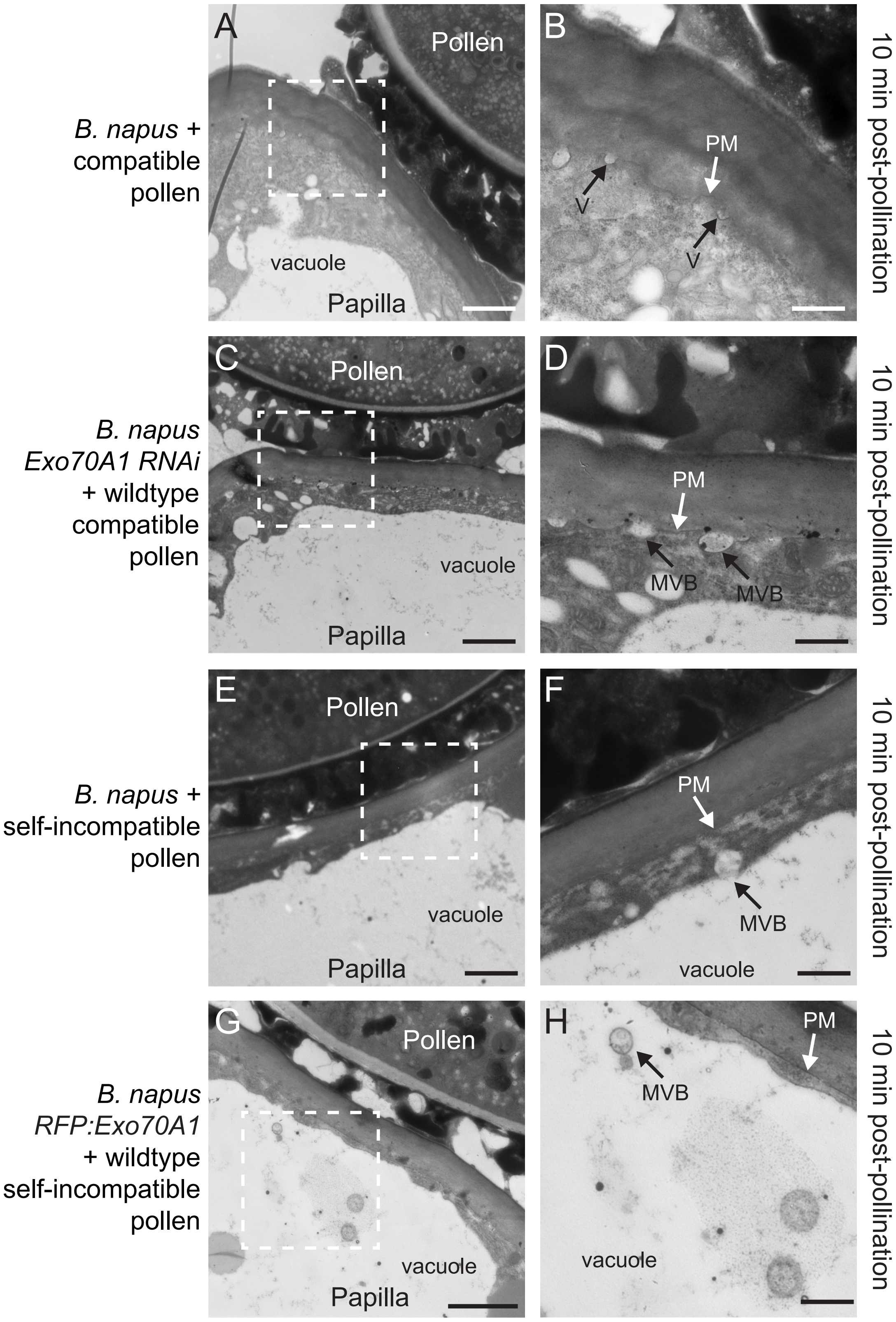

Figure S2. TEM images of B. napus stigmatic papillae showing alternate trafficking patterns.
(A, B) *B. napus* Westar stigmatic papilla at 10 min post-pollination with self-compatible pollen. Vesicles (V) appear to be fusing to the papillar plasma membrane (PM) underneath the pollen contact site in 2/25 samples.
(C, D) *B. napus* Westar *Exo70A1* RNAi stigmatic papilla at 10 min post-pollination with wild-type compatible pollen. MVBs appear to fuse with the plasma membrane (PM) in 2/10 samples. This observation is consistent with the incomplete knockout phenotype of the *B. napus* Westar *Exo70A1* RNAi plants [17].
(E, F) *B. napus* W1 stigmatic papilla at 10 min post-pollination with self-incompatible pollen. MVBs appear to be targeted to the vacuole after self-incompatible pollinations. These images show a potential fusion of a MVB to the vacuole, and intact MVBs were not visible inside the vacuole as observed in 3/10 samples.
(G, H) *B. napus RFP:Exo70A1* W1 stigmatic papilla at 10 min post-pollination with self-incompatible pollen. MVBs were observed in the vacuole in 2/10 samples which is consistent with these plants displaying an incomplete self-incompatibility response [17].
The white boxed areas in (A, C, E, G) are shown in the (B, D, F, H), respectively. Scale bars (A, C, E, G) 1.5 µm; (B, D, F, H) 500 nm.
